# Certification of SRM1960: Nominal 10 μm Diameter Polystyrene Spheres (“Space Beads”)

**DOI:** 10.6028/jres.096.044

**Published:** 1991

**Authors:** Thomas R. Lettieri, Arie W. Hartman, Gary G. Hembree, Egon Marx

**Affiliations:** National Institute of Standards and Technology, Gaithersburg, MD 20899

**Keywords:** Electron microscopy, length, light scattering, metrology, microgravity, micrometrology, microscopy, microspheres, particles, particle sizing, polystyrene spheres, reference materials, sizing, standards

## Abstract

Experimental, theoretical, and calculational details are presented for the three independent micrometrology techniques used to certify the mean diameter of Standard Reference Materisd 1960, nominal 10 μm diameter polystyrene spheres (“space beads”). The mean diameters determined by the three techniques agreed remarkably well, with all measurements within 0.1% of each other, an unprecedented achievement in the dimensional metrology of microspheres. Center distance finding (CDF), a method based on optical microscopy, gave a value of 9.89 ± 0.04 μm, which was chosen to be the certified mean diameter. The supporting measurements were done using metrology electron microscopy (MEM) and resonance light scattering (RLS). The MEM technique, based on scanning electron microscopy, yielded 9.89±0.06 μm for the mean diameter of the microspheres in vacuum, while the RLS value was 9.90 ±0.03 μm for the microspheres in liquid suspension. The main peak of the diameter distribution for SRM 1960 is nearly Gaussian with a certified standard deviation of 0.09 μm, as determined by CDF. Off the main peak, there are about 1% oversized particles and a negligible amount of undersized particles. The report gives a detailed description of the apparatus, the experimental methods, the data-reduction techniques, and an error analysis for each of the micro-metrology techniques. A distinctive characteristic of this SRM is that it was manufactured in microgravity aboard the NASA space shuttle Challenger and is the first commercial product to be made in space.

## 1. Introduction

The National Institute of Standards and Technology (NIST), in a cooperative effort with ASTM, has completed the certification of a series of monodisperse particle-sizing Standard Reference Materials (SRMs) for use in instrument calibration and as benchmark standards for microdimensional metrology [1,2]. Six SRMs are now available: SRM 1691 (nominal 0.3 μm spheres); SRM 1690 (nominal 1 μm spheres); SRM 1962 (nominal 3 μm spheres); SRM 1960 (nominal 10 μm spheres); SRM 1961 (nominal 30 μm spheres); and SRM 1965 (a microscope slide containing the nominal 10 μm spheres).

**Fig. 1:**
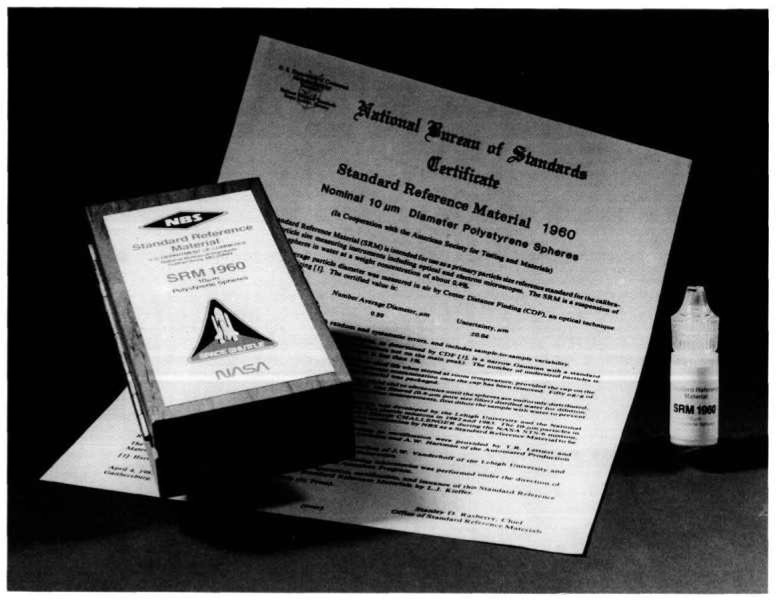
Photo of NIST Standard Reference Material 1960 showing a vial of the SRM, the certificate, and the package.

**Fig. 2:**
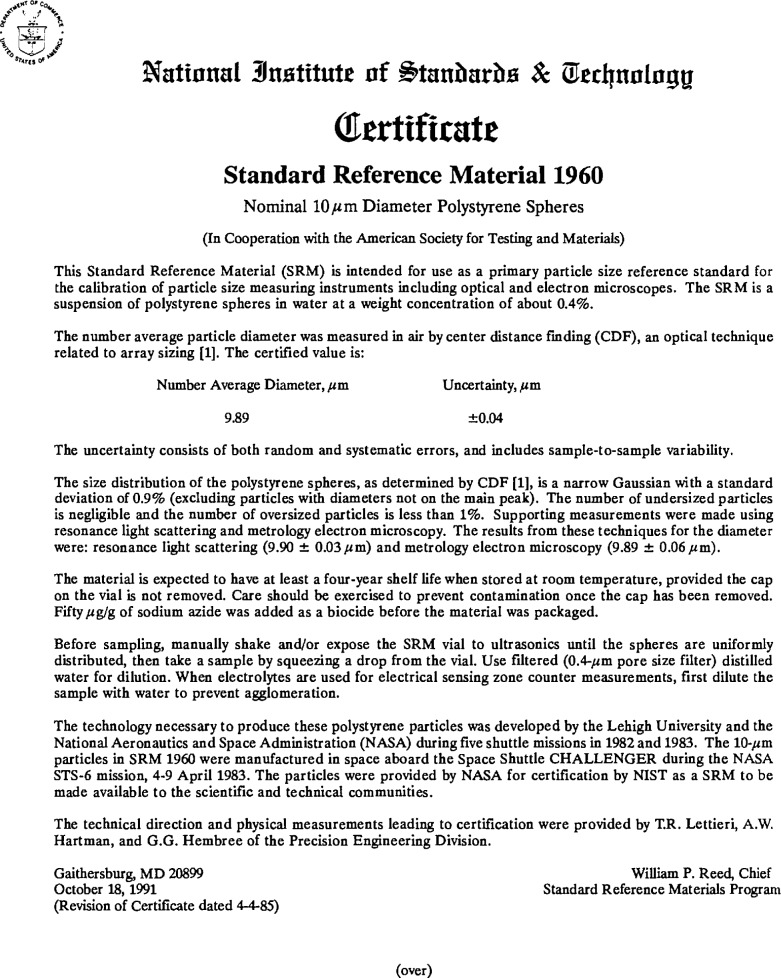

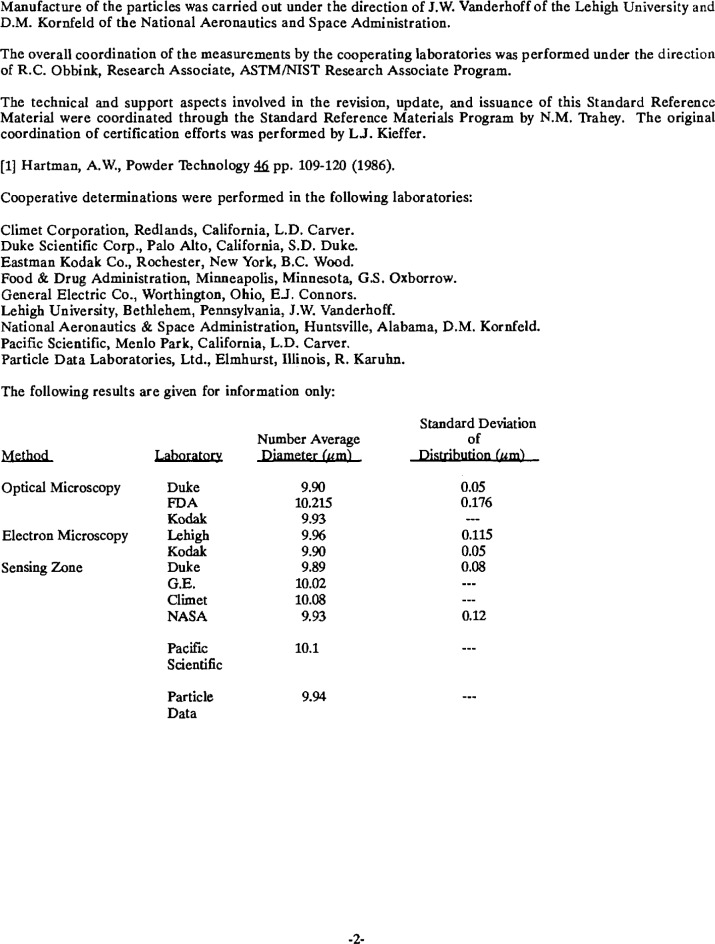
The certificate which comes with SRM 1960. The certificate which comes with SRM 1960 (reverse).

**Fig. 3:**
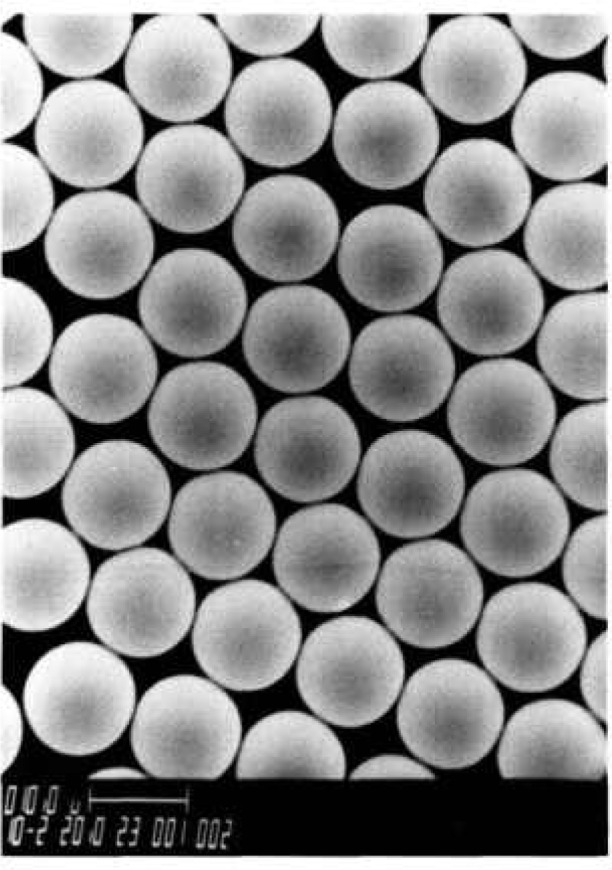
Scanning electron microscope photomicrograph of the SRM 1960 microspheres.

The present report describes the certification process for SRM 1960, nominal 10 μm diameter spheres (Figs. 1, 2, and 3). Three micrometrology techniques were used to get an accurate mean diameter for these polystyrene microspheres [3]: center distance finding (CDF), metrology electron microscopy (MEM), and resonance light scattering (RLS). The results from each technique agreed to well within the stated uncertainties, with the CDF value of 9.89 ±0.04 μm assigned as the certified mean diameter. In addition, CDF was used to get a certified value of 0.09 μm for the standard deviation of the main peak of the size distribution.

SRM 1960 is packaged in 5 ml plastic vials at a weight concentration of particles of 0.4% (Fig. 1); there are thus about 40 million particles in each vial. To prevent the growth of biological organisms, 50 ppm of sodium azide were added as a biocide before the material was packaged. The material is also available on a microscope slide (SRM 1965) for calibrating optical microscopes, among other uses in micrometrology [4]. The SRM 1960 spheres were grown in a microgravity environment aboard the NASA space shuttle Challenger during its STS-6 mission in April 1983, making this SRM the first product to be made in space for commercial use [5]. Details of the polymerization processes used to grow the microspheres are given elsewhere [6].

In this report, the experimental, theoretical, and calculational procedures for each technique, and their sources of uncertainty, are discussed in detail. The center distance finding technique is described first, followed by descriptions of metrology electron microscopy and resonance light scattering.

## 2. Center Distance Finding

The certified diameter for SRM 1960 was determined using center distance finding (CDF). This micromeasurement technique uses a conventional optical microscope and has the advantages of high resolution (0.03 μm, comparable to that of electron microscopy), high accuracy (the image magnification of an optical microscope is a stable, well known number), and a non-harsh environment (no vacuum, no electron beam irradiation). For these reasons, and because of the high accuracy of the technique, the CDF values for the mean diameter and the size distribution width were the ones chosen to be the certified values for the SRM.

### 2.1 Experimental Approach

**Fig. 4:**
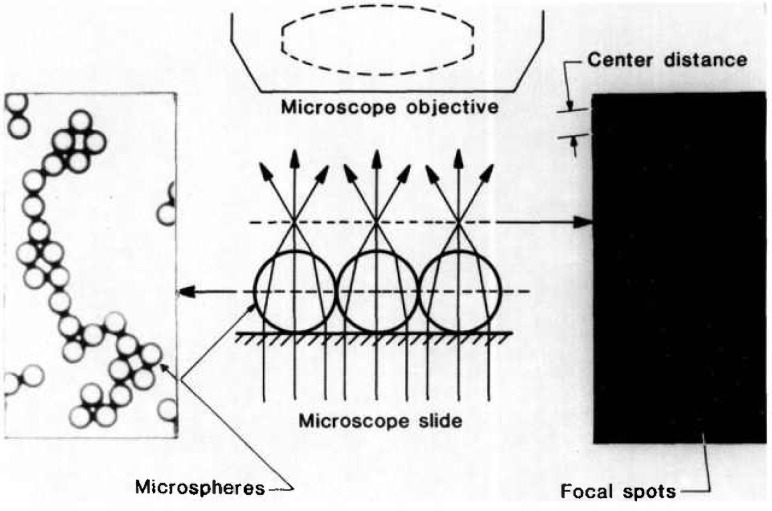
The center distance finding (CDF) technique.

In the CDF technique, the microspheres are arranged on a microscope slide as two-dimensional contacting structures, which are then illuminated with parallel light (Fig. 4). In this configuration, each transparent microsphere acts like a positive lens and refracts the transmitted light into a small, circular focal spot (Fig. 4). These spots mark the locations of the microsphere centers, and the center distances (CDs) between contacting spheres contain the diameter information that is desired. The dot patterns are then photographed and the CDs measured from the photographic film and converted into distances in the object plane, using accurately known values for the optical image magnification on-axis and elsewhere in the field of view (FOV). This procedure is much more precise than the microsphere edge detection used in conventional array sizing [7].

If, as is often the case with monosize microsphere materials, the particles have a Gaussian size distribution, then the CDs will also be normally distributed with a standard deviation that is $\sqrt{2}$ times smaller (because each CD averages over the diameters of two spheres [8]). Conversely, when the measured microsphere material exhibits a Gaussian CD distribution, both the mean diameter and the diameter distribution can be deduced. This can be done with an uncertainty much smaller than the wavelength of the light used in the microscope.

**Fig. 5:**
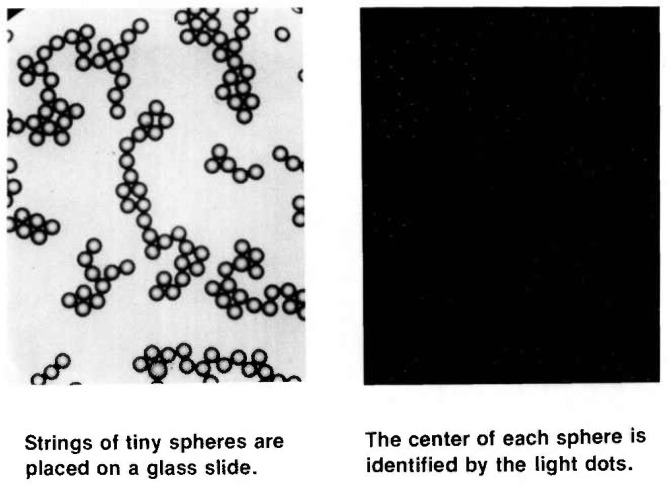
CDF disordered assemblies.

In the CDF measurements of SRM 1960, the microsphere structures were not the usual hexagonal arrays, but instead were disordered assemblies (Fig. 5). These are used in order to avoid measurement errors caused by air gaps between spheres [9]; such air gaps are natural to hexagonal arrays and will lead to errors in the CDF measurements (see “CDF Error Analysis” section).

**Fig. 6:**
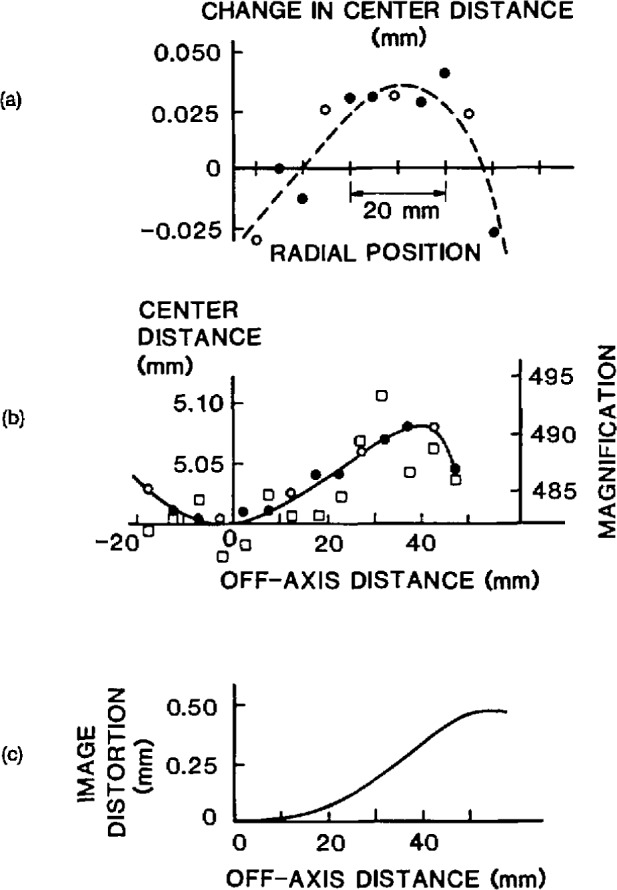
Microscope calibration curves for image magnification and distortion.

Since the CDF technique relies on accurate measurements of sphere centers in photomicrographs, the film scale must be well known everywhere in the FOV used, and the dimensional stability of the photographic film must be sufficient to support these measurements. Thus, a precision calibration of the microscope for image magnification everywhere in the utilized FOV is an essential part of the microsphere diameter-distribution measurement by CDF. The procedure used to calibrate the microscope for image magnification is described in the Appendix. Figure 6 shows the magnification values vs. off-axis distances determined using this calibration procedure [8]. The area on the film that was used in the measurements had a diameter of about 80% of the short dimension of the 100 × 125 mm film (i.e., about 80 mm in diameter).

### 2.2 Experimental Method

Six samples of microspheres (C1–C6) were taken from four different vials of SRM 1960 and then diluted from 0.4% weight concentration to about 0.1% weight concentration using ultra-pure, 25 MΩ cm water. For each sample, a drop of the diluted suspension was placed on a microscope slide, spread out, and then allowed to dry. Sparse and disordered structures (much like strands of beads) formed, in which most spheres had only one or two contacting neighbors. In such structures, air gaps, which would cause measurement errors, are unlikely to develop [7].

The microscope slide was then illuminated by parallel, quasi-monochromatic light approximated by stopping the microscope condenser all the way down and putting a green filter into the beam. The parallel light was focussed by the individual microspheres to a common back focal plane. Photomicrographs taken of the focal plane showed focal-spot patterns which corresponded to the microsphere structures (Fig. 5). The focal spots were small and circular, about 0.5–1.0 μm in diameter in the object plane, the smallest spots (0.5 μm diameter) being obtained by a judicious choice of film material and exposure time.

The film scale was chosen to be large enough that the distances between focal spots could be measured with a resolution of about 1 part in 300 to 1 part in 500, but not so large that excessive numbers of photographs had to be taken to cover a measured sample of about 2000 spheres, in total. Such a large sample size was needed to get an accurate value for the standard deviation. If only the mean diameter were desired, then about 200 spheres would have been enough. A useful film scale is 500 ×, giving 5 mm CDs in the photomicrographs.

For reasons of speed and convenience, Polaroid^2^ Type 57 (3000 ASA) positive film was used. This material has low graininess, and the dimensional stability is adequate for the CDF measurements [8], Focal-spot spacings on the film were measured automatically in a coordinate measuring machine (CMM) using a low-power (30 ×) microscope as the probe. To make a CD measurement, the CMM microscope cross hairs were centered on a focal spot, and the *x-y* coordinates of the spot were automatically entered into computer memory at the push of a button. To decrease the effect of film graininess, the microscope was slightly defocused, enabling an experienced observer to visually pinpoint the center of each spot, which typically had a diameter of about 0,3 mm, to a precision of 0.01 mm (or 0,02 μm in the object plane). A computer program then calculated the CD spacings. In this manner, sphere CDs were found with a precision of about ± 0.03 μm.

### 2.3 CDF Results

**Fig. 7:**
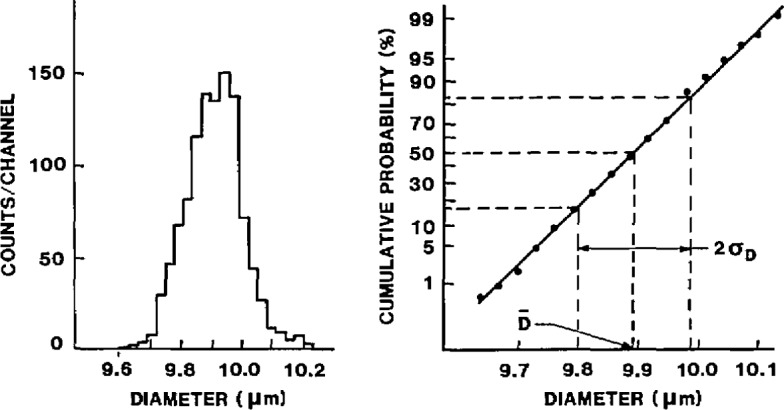
Diameter distribution of the SRM 1960 spheres, as determined using CDF.

The data taken from the photographic film were distances, *c*, between sphere centers. Using the above CDF procedures, *c*-values were measured, and the *c*-distribution was plotted and verified for normality. Then, the mean diameter (*d*_m_) and the standard deviation (*σ*_d_) of the microspheres were determined using *d*_m_ = *c*_m_ and $\sigma_{d}=\sqrt{2\times \sigma_{\mathrm{cd}}}$, where *σ*_cd_ is the standard deviation of the center distance measurements. The results of the CDF measurements on SRM 1960 are given in Table 1, and the diameter distribution is shown in Fig. 7.

A useful consequence of the CDF technique is that the sphericity of the individual particles can be determined by looking at the shape of the photographed focal spots. In general, the focal spots were visually of circular shape, with occasional (<1%) shapes that were elongated by 5–10% or more. Considering that the elongation of the focal spot is the same as that of the sphere, it can be concluded that the vast majority of SRM 1960 microspheres has an asphericity amounting to less than 0.5% (as measured perpendicular to the line of sight). In short, these particles are very close to being perfect spheres.

### 2.4 CDF Error Analysis

As with all measurements, both random and systematic errors occurred in the CDF measurements described above. The major random errors were center-finding uncertainty, film instability, magnification scatter, and small sample size (sampling error). The primary sources of systematic error were image magnification error, image distortion error, and sphere flattening. Uncertainties due to air gaps and foreign material between microspheres were determined to be negligible.

Note that in all of the error analyses below (for CDF, MEM, and RLS), the random errors are given as 3*σ* (99% confidence level). In addition, all of the random uncertainties contain a component due to vial-to-vial variability, if present, since particles from several vials were measured by each technique.

#### 2.4.1 CDF Random Errors

An estimate of the random error in the CDF measurements can be obtained by finding the 3*σ* of the five valid diameter measurements in Table 1. For these measurements the 3*σ* random uncertainty, *R*, is calculated to be ±0.0047 μm; this is the value of the random error used later to calculate the total uncertainty [Eq. (3)]. In addition, it is useful to determine the sources of the various CDF random errors and calculate estimates for their individual contributions. These error sources are discussed below.

##### Center-Finding Uncertainty and Film Instability

These two errors limit the ability to reproducibly locate the center of a given focal spot on the photograph. The cross hair in the probe microscope of the CMM was placed over the center of a photographed focal spot, guided by the eye of a trained observer. As noted earlier, the probe microscope was slightly defocused in order to reduce the effect of film graininess. This centering process is limited by the acuity of the eye and its sensitivity to rotational symmetry. It is also affected by the dimensional stability of the film: photographic emulsions are known to shift laterally after exposure due to film developing, fixing, and drying. The combined effect of these two sources is a scatter in the measured *X–Y* coordinates of a focal spot when photographed and measured under identical circumstances. This (combined) error contribution to single measurements of CDs is a random one and was measured as follows. A row containing 16 microspheres was centered in the FOV, and its corresponding row of focal spots was photographed five times; all CDs between adjacent spheres in the row were measured in each photograph. The data obtained were scaled such that all sets of five CDs had the same average value. This removed the effects of unequal sphere size and of any spurious changes in magnification associated with the process of taking repeated photographs. The result was a pooled set of 75 CDs. When analyzed, the data showed a 3*σ* scatter of 38 μm in a single measurement of a 5 mm CD (0.075 μm in the object plane). As measured earlier [8], the dimensional stability of the Polaroid film is known to be about 10 Jim across the entire film.

Assuming these two sources of error combine in quadrature, the CD uncertainty contribution from the focal-spot-pinpointing process is slightly less than 40 μm. This corresponds to a ± 0.08 μm random error per CD measurement in the object plane.

##### Magnification Scatter

When the microscope is refocused between exposures, the object distance changes somewhat: the final image shifts along the optical axis, its distance to the photo eyepiece changes, and the magnification varies accordingly. However, if the film plane is held fixed, the image scale in the film remains constant (to first approximation), although the image loses sharpness.

The situation changes when fresh film is inserted into the cassette. The flexible film sheet is held by its edges, no vacuum platen is used, and the film plane can change in axial position by some 0.1 to 0.2 mm. The distance to the photo eyepiece is typically 150 mm, hence spurious changes in film scale can be expected at the 0.1% level. These changes were measured as follows.

**Fig. 8:**
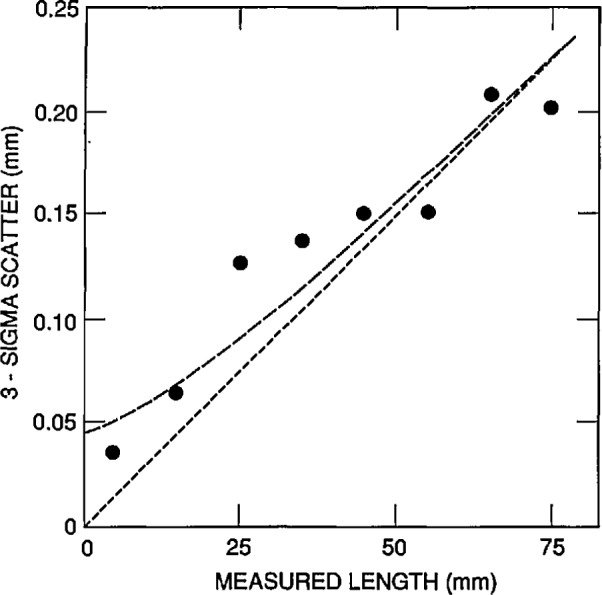
Scatter in the microscope magnification.

Using the five photographs mentioned earlier, the lengths of row sections containing 1,2,3,4, etc., CDs were measured, up to the full FOV. For each set of five nominally equal lengths, the length scatter was found and plotted against the length itself. The plot shown in Fig. 8 approaches, for large lengths, a straight line passing through the origin. The slope of this line shows that part of the total length scatter is proportional to length and, thus, is caused by fluctuations in magnification. These amoimted to a 0.2%, or ±0.02 μm, random error when measuring 10 μm lengths.

##### Sampling Error

The sampling error, due to the finite sample chosen from a large population of microspheres, is given by:

$$r=t_{m}(0.005)\sigma_{d}/\sqrt{n}$$ (1)

in which *σ*_d_ is the standard deviation of the diameter distribution, *t_m_* is the Student *t*-value for *m* degrees of freedom at the 99% confidence level and *n* ≈ 2000 is the number of microspheres sized by CDF (the value of *m* is one less than this). Substituting into Eq. (1) gives a value of 0.27 μm sampling error for a single measurement of *d*_m_, or ±0.006 μm for all 2000 measurements.

##### Total Random Error

Summing the above contributions in quadrature gives a total random uncertainty of ± 0.006 μm, in good agreement with the 3*σ* random uncertainty (± 0.0047 μm) calculated from the five CDF measurements.

#### 2.4.2 CDF Systematic Errors

##### Image Magnification and Distortion Errors

The section of the stage micrometer that was imaged had a length of 160 μm and had been calibrated at NIST with ±0.04 μm accuracy using a photoelectric image scanner and traveling stage with interferometric readout. This amounts to a systematic error of ±0.003 μm when measuring 10 μm distances. The microscope image of the stage micrometer was 90 mm long, measured with an uncertainty of 0.05 mm, resulting in a systematic error contribution of ±0,006 μm.

To account for image distortion, the uncertainty in the length correction of the micrometer image was 0.05 mm, giving a ±0.006 μm systematic error on the mean diameter. The micrometer image length was found by taking 5 repeated exposures, thus reducing the magnification scatter to 0.09% (compared to 0.2% for a single exposure) giving a ± 0.009 μm systematic error in the microsphere diameter measurement.

The scale distortion in Fig. 6b is found with an uncertainty of 0.01 mm, or 0.2%. The image magnification as a function of off-axis distance is thus known to about 0.3%. The scale-distortion relationship could have been entered into the computer-automated, CD-measuring algorithm as a lookup table. Here, however, the distortion data was used to make a transparent overlay that was placed over each measured piece of film. The overlay consisted of a series of concentric zones marked with appropriate corrections for measured CDs. The use of an overlay adds an estimated ±0.01 μm systematic error and a ± 0.02 μm random error, when measuring 10 μm lengths anywhere across the FOV.

##### Sphere Flattening

The primary forces which adhere small particles (diameter < 50 μm) to dry surfaces are van der Waals forces. During drying, strong capillary forces act on the microspheres, bringing them into intimate contact [9], Since the polystyrene spheres of SRM 1960 have optically smooth surfaces, the van der Waals attraction at the initial area of contact will pull adjacent areas into contact. This phenomenon is resisted by elastic sphere deformation.

Muller et al. [10] have analyzed the balance between these two processes. They give expressions for the flattening of spheres in contact with a flat substrate and show the sphere deformation at and around the contact area, indicating that the active, non-contacting zone is relatively small when no external forces are present. In that case, the flattening of a sphere contacting an equal-size sphere will be essentially equal to that of a sphere contacting a plane, and the Muller expression can be doubled to find the decrease in sphere CD due to the van der Waals attraction. The diameter correction for sphere flattening will then be:

$$\Delta d_{f}=+\frac{1}{4}{\left(\frac{3{\left(1-v^{2}\right)}^{2}rA^{2}}{2E^{2}\epsilon^{4}}\right)}^{1/3},$$ (2)

in which *v* = the Poisson constant = 0.3 for polystyrene; *r* = sphere radius = 5 μm; *A* = Hamaker constant = 1 × 10^−12^ erg for polystyrene surfaces; *E*= Young’s modulus = 3×10^10^ dyn/cm^2^ for polystyrene; and *ϵ* = closest distance of approach = 0.3 nm. Substituting these values into Eq. (2) gives a systematic diameter correction due to van der Waals attraction amounting to + 0.002 μm.

##### Air Gaps

Air gaps between spheres, if present, would result in an overestimate of the mean diameter [7]. Gaps wider than about 0.2 μm can be found by visual inspection of the microsphere images. Narrower gaps can be detected in selected, sparse structures that are arranged as chains or strands and which contain a triangular sphere arrangement at two or more locations (Fig. 5). Measuring one triangle yields three radii. Sphere diameters farther along the chain are found from CD measurements between known and unknown spheres, until the whole group is measured. The process is then repeated, starting from another triangle. The result is two sets of sphere diameters and, if these are equal within experimental limits (i.e., if there is closure), the air gaps can be assumed absent in that structure. The measured chain should preferably be short, to reduce measurement error accumulation. Spot checks like this in the microsphere preparation confirmed the likely absence of air gaps in the CDF measurements reported here.

Another indication of the absence of air gaps could be found by observing the sphere-grouping process during drying of the deposited microspheres. Just before final drying, spheres which had already attached themselves to the substrate were seen to be pulled toward each other in a “snap-like” fashion, giving the impression that they were torn loose from the substrate by a water film acting like a stretched elastic membrane. This mechanism is unlikely to result in air gaps.

Thus, for purposes of the present calibration process, air gaps were assumed to be absent in the measured microsphere structures.

##### Foreign Material

Since the bottled SRM 1960 suspension contains 50 ppm of a biocide (sodium azide), it is possible that a surface coating of this foreign material can cause an overestimate of the mean diameter. In the CDF measurements, the microspheres covered about 5% of the glass slide area. Given the 0.4% weight concentration of particles in the bottled SRM, if all of the fungicide stays behind after drying as a hard, uniform layer coating both the spheres and the slide, this coating will add, at most, 0.0001 × *d*_m_ to the measured diameter of the spheres. Such a small correction can be safely neglected.

In addition, dilution by one and two orders of magnitude did not change the measured mean diameter, suggesting that any coating between the spheres is punctured in the last moments of drying. This is also indicated by the behavior of the drying spheres, which snap together into intimate contact. For the purpose of the SRM 1960 certification by CDF, it is therefore assumed that no foreign material is present between the dried spheres.

#### 2.4.3 Total Diameter Uncertainty from CDF

All of the contributions to the CDF measurement uncertainty are summarized in the error budget in Table 2. The total error is given by [11]

$$\begin{array}{l} U_{T}=R+U_{S}\\ \;=R+|\delta_{m}|+|\delta_{d}| \end{array}$$ (3)

in which *R* is the total random error = 0.0047 μm. *U*_S_ is the total systematic error, *δ*_m_ is the systematic image-magnification error and *δ*_d_ is the systematic image-distortion error. Substituting the various error values into Eq. (3) gives a total uncertainty of ±0.04 μm for the CDF measurements.

### 2.5 Final Results of the CDF Measurements

The final results for the CDF calibration are: *d*_m_ = 9.89±0;04 μm and *σ*_d_ = 0.09 ± 0.01 μm. The diameter uniformity within vial and between vials is ±0.1%. The microsphere diameter distribution was found to be normal (Gaussian) from 1% to 99%. The number of outliers found by visual inspection (i.e., finding spheres with diameters clearly outside the main peak, by 0.05 × *d*_m_ or more) is approximately 1% for oversized particles and negligible for undersized particles.

## 3. Metrology Electron Microscopy

A supporting technique used in the measurement of the SRM 1960 microspheres was metrology electron microscopy (MEM). The value of the MEM technique is that it ties the dimensional measurements of the microspheres to the wave-length of a stabilized helium-neon laser, a widely used secondary length standard. In addition, the technique provides a check for possible systematic errors in the other techniques which may be due to environmental factors: for CDF, the particles are measured dry in air, while for RLS they are measured in a liquid environment. In contrast, the MEM measurements are made on the microspheres in an ultra-high vacuum, providing a test for possible dimensional instability and/or out-gassing of the polystyrene particles.

### 3.1 Experimental Apparatus

**Fig 9:**
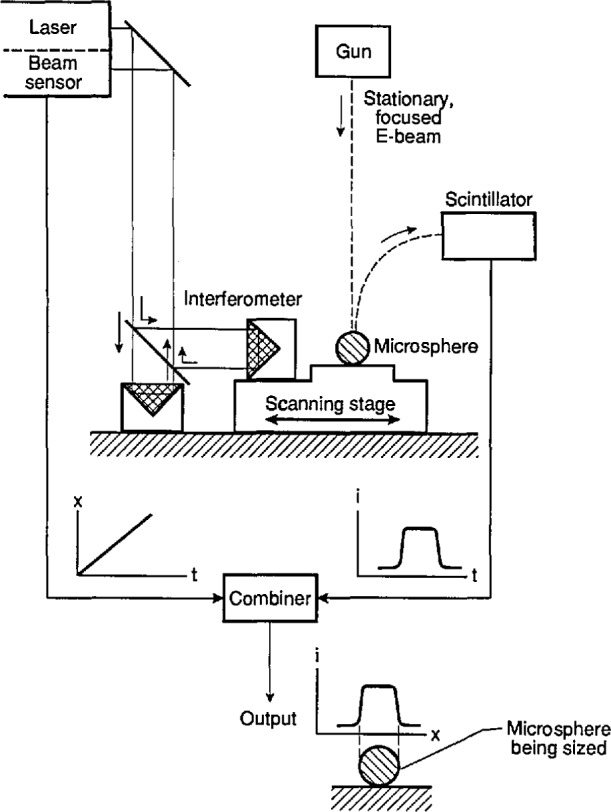
Schematic diagram of the metrology electron microscope (MEM) system.

The MEM system is based on a commercial ultra-high vacuum scanning electron microscope (SEM) with a field-emission electron gun [12]. In the MEM, the electron beam is fixed in position, so that it acts as a reference point or cross hair. The microsphere is then translated through the e-beam using an electro-mechanically scanned stage (Fig. 9). Displacement of the stage is monitored by a commercial heterodyne interferometer system which uses a stabilized helium-neon laser to set the metric [12]. In this way, the MEM measurement of the microsphere diameter is directly tied to the wavelength of the helium-neon laser (≈632.8 nm).

The MEM stage uses a piezoflex driving element whose displacement is magnified by two sets of flexure-pivot lever arms [13]. The stage is fabricated from a single piece of 304 stainless steel and has a maximum displacement of 170 μm. Roll, pitch, and yaw are all less than 2 arcsec with 3 kV applied voltage on the piezo-electric transducer (PZT). With the piezoflex stage, the displacement of the microspheres across the electron beam is as smooth as the applied voltage down to the subnanometer level [13].

As the particle is scanned through the e-beam, the position of the stage is monitored by a heterodyne fringe-counting interferometer; for the SRM 1960 microspheres, the scan time across a particle was about 10 s. The interferometer is a single-pass Michelson type with a polarizing beamsplitter and glass retroreflectors [14]. The measurement retroreflector is removable to allow alignment of the stage interferometer before installation in the microscope. To minimize dead-path errors, the reference and measurement optical paths are made equal in the interferometer arrangement. In operation, the laser beam enters and exits the beamsplitter through a window on top of the vacuum chamber of the SEM (Fig. 9).

**Fig. 10:**
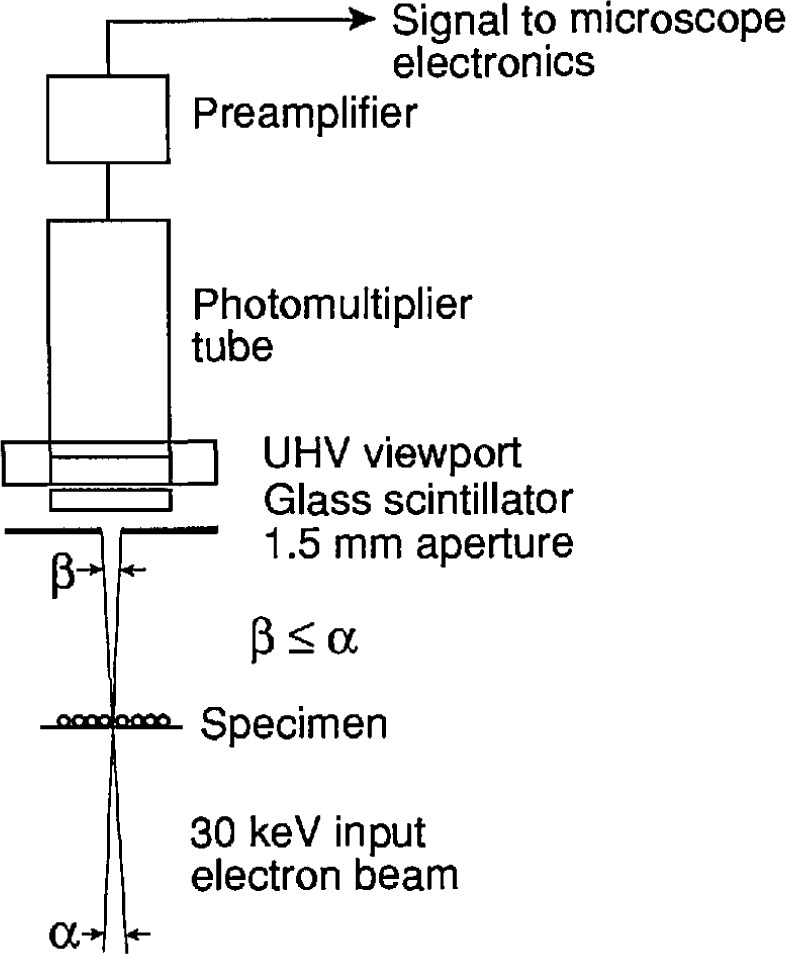
Schematic diagram of the bright-field imaging mode in the MEM.

A bright-field transmission detector was employed to collect the intensity profile while a particle was being scanned. As shown in Fig. 10, this detector consists of a small aperture placed in front of a scintillation detector. When the angular size (*β*) of the aperture, as measured from the specimen, is equal to or smaller than the angular size (*α*) of the electron beam, the detector will only produce a significant signal if the beam does not scatter from the specimen [12]. In the present case, the aperture was about 1.5 mm in diameter, which corresponds to an angular size of 3.5 mrad. At 30 keV beam energy and a working distance of 25 mm, this size matches that of a 10 nm electron probe. Beam current in the measurements was about 0.5 nA.

**Fig. 11:**
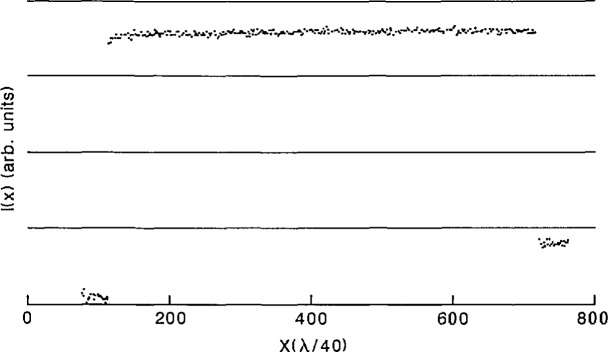
MEM intensity profile of an SRM 1960 microsphere.

After a particle was scanned, a computer analysis of the electron-intensity profile gave its measured diameter. Since the edge resolution under the above noted e-beam conditions was less than the interferometer resolution of 16 nm, the edge-detection algorithm in the computer code could easily locate the transition point from the particle to the background. The algorithm determined the edges of a particle by calculating a separate threshold for each edge based on 10% of the total rise or fall from the background level (Fig. 11).

### 3.2 Experimental Method

The SRM 1960 samples were prepared for the MEM by diluting one drop from a vial into 50 ml of 18 MΩ cm deionized water and then ultrasonicating, settling, and decanting 80% of the supernatant liquid. This washing cycle was repeated three times for each sample to remove as much of the water-soluble additives as possible. A small drop of the washed suspension was dried down onto a thin carbon foil supported by a 200-mesh copper TEM grid and then overcoated with about 20 nm of amorphous carbon in a vacuum evaporator to minimize charging in the electron beam. After overcoating, the grids were loaded into the MEM chamber, which was pumped down to a 10^−8^ Torr vacuum.

Three different vials of SRM 1960 were sampled, and one grid was prepared from each sample (these are labelled M1, M2, and M3). About 30 individual microspheres were measured on each grid to give good statistics on the mean-diameter determination; this was not enough particles, however, to get an accurate measure of the standard deviation. Visibly obvious outliers were not included in the measurements.

The computer-based data acquisition system was programmed to set up a scan and then pause between each diameter measurement to allow the operator to locate, manually position, and then focus on each particle to be measured. After the operator switched the MEM to spot mode and restarted the measurement computer program, the program controlled the stage scan, collected the data on intensity vs. stage position, calculated the particle diameter, and reported the measured diameter value. After all of the particles in one sample were measured, the computer program calculated the mean diameter.

For the first sample (M1), all of the microspheres were scanned three times to determine the amount of particle shrinkage due to e-beam irradiation; typically, about 3% shrinkage was measured after the three scans (see Table 3). To avoid this problem, only the first particle scan was used for all of the MEM measurements.

### 3.3 MEM Results

A typical trace of the inverted bright-field intensity profile for a single SRM 1960 microsphere is shown in Fig. 11. The intensity was sampled at 500 points, equally spaced in time, and the stage position was recorded simultaneously. The total scan length was approximately 10.6 μm; therefore, each sampled point corresponds to about 20 nm in stage displacement. As the profile in Fig. 11 indicates, the transition at the edges is sharp to within one sampled point, making the edge-detection algorithm relatively straightforward, as noted.

A summary of the results for the three samples, labelled M1, M2, and M3, are presented in Table 4. Several measurements in each sample were discarded as being outliers, either over- or undersized, as determined by a discordancy test based on the sample kurtosis [15]. In each case, the outlier was more than 3*σ* away from the mean, either lower or higher in diameter. The final reported number-average mean diameter, *d*_m_, is taken to be the mean value of the three independent measurements, 9.886 μm.

### 3.4 MEM Error Analysis

Random errors are the major source of uncertainty in the MEM measurements, the primary ones being sampling error, spatial resolution and (random) e-beam wander, and cosine error. The systematic uncertainties include least-count in the interferometer, digitization of stage travel, and e-beam erosion of the microspheres. Potential error sources that were determined to be negligible in the MEM measurements were due to particle outgassing in a vacuum, carbon coating on the particles, and interferometer error.

#### 3.4.1 Random MEM Errors

An estimate of the random error in the MEM results was obtained by finding the 3*σ* of the data in Table 4. This value, ± 0.018 μm, was used in the calculation of the total MEM error,

$$\begin{array}{l} U_{T}=R+U_{S}\\ \;=R+|\delta_{\mathrm{lc}}|+|\delta_{d}|+|\delta_{e}|. \end{array}$$ (4)

Possible sources of this random error are summarized below.

##### Sampling

As with the CDF measurements, the sampling error arises from the limited number of microspheres measured, as taken from a population with a finite size distribution having a standard deviation, *σ*_d_, of 0.9% of the mean diameter. Using Eq. (1) with *n* = 81, a value of ±0.03 μm is obtained for the MEM sampling error.

##### Spatial Resolution and e-Beam Wander

The point-to-point resolution of the scanning electron microscope used in the MEM measurements, essentially due to finite spot size and random beam wander, was measured to be ±0.02 μm. Since two microsphere edges have to be detected, the random uncertainty is $\sqrt{2}$ times this or 0.028 μm per measurement. The random error for 81 measurements is thus $0.028/\sqrt{81}=0.003$ μm.

##### Cosine Error

Cosine error occurs in the MEM if the microsphere is not measured along the diameter but, rather, along a chord of the projected sphere image. This error is expected to be small, since it is easy to visually determine the diameter of a circle to better than 1 part in 30. Using the expression for cosine error,

$$\delta_{c}\approx d_{m}(1-\cos\alpha )\approx d_{m}(\alpha^{2}/2)$$ (5)

in which *d*_m_ is the mean diameter of the microspheres and *α* is the scan-angle error, this uncertainty was determined to be at most 0.014 μm per measurement, assuming *α* ⩽3°. Since this is a one-sided error, the random error for all 81 measurements is approximately 0.014/4 = 0.003 μm.

##### Total Random Error

Combining the above three components in quadrature gives a total random error of ±0.03 μm, somewhat higher than the *R* =0.018 μm determined from the three MEM measurements of the mean diameter.

#### 3.4.2 Systematic MEM Errors

##### Least Count in Interferometer

The least-count systematic uncertainty in MEM is due to the inability to determine the intensity-transition point in the microsphere scans to better than the least count of the interferometer, which is *λ*/40 = 16 nm (Fig. 11). Since two transitions must be determined (one on either side of the particle), this error is equal to twice the halfwidth of the sampled point, or ± 0.016 μm.

##### Digitization of Stage Travel

The MEM stage travel of 10.6 μm was sampled at 500 equidistant points, resulting in a 10.6/500 μm=0.02 μm systematic error on the measurement of stage displacement.

##### E-Beam Erosion of the Microspheres

This error arises from erosion of the SRM 1960 microspheres as they pass through the e-beam. To minimize this effect, only the first MEM scan of a particle was used to determine the mean diameter. Nevertheless, there will still be some residual particle shrinkage for one scan. The magnitude of the shrinkage was determined by repeatedly scanning across the same line on a microsphere; this was done for ≈60 microspheres in sample M1. Typical results for three scans of 5 microspheres measured sequentially from this sample are given in Table 3. From all of the measurements, the decrease in particle diameter due to electron irradiation was determined to be about 0.1% per scan, or about a ± 0.01 μm systematic error in the mean diameter.

#### 3.4.3 Total Diameter Uncertainty from MEM

All of the MEM errors are summarized in Table 5. As specified by Eq. (3), these errors are combined as Eq. (4), which gives the total uncertainty on the MEM measurement as ± 0.06 μm. In Eq. (4), *δ*_lc_ is the least-count error, *δ*_d_ is the stage digitization error, and *δ*_e_ is the e-beam erosion error.

### 3.5 Final Results of the MEM Measurements

The mean diameter of SRM 1960 determined from metrology electron microscopy is 9.89 ±0.06 μm.

## 4. Resonance Light Scattering

The third technique used in the certification of SRM 1960 was resonance light scattering (RLS). This method uses the sharp resonances which occur in the Mie light scattering cross-sections of dielectric microspheres as a function of incident light frequency [16]. Resonance light scattering spectra can be used to accurately determine the diameter of a single microsphere by quantitatively comparing the experimental resonance wavelengths with those calculated from a Mie scattering model [17]. In principle, a sufficient number of SRM 1960 particles could have been individually measured in this manner to build up the size distribution. However, in the RLS experiments described in the present report, a simpler method was used whereby a single RLS spectrum is measured from a large number of microspheres in liquid suspension [18]. The peaks in this collective spectrum are broader than those in single-particle spectra, but are still sharp enough to yield high-resolution diameter information.

### 4.1 Experimental Apparatus

**Fig. 12:**
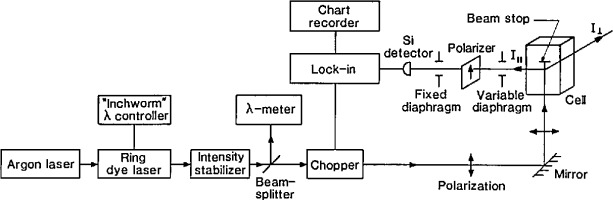
Schematic of the resonance light scattering (RLS) apparatus.

The experimental RLS apparatus is diagramed in Fig. 12 [18]. The ring dye laser was pumped by an argon-ion laser, and its intensity was stabilized by an electro-optical feedback system. The beam was vertically incident into a glass sample cell filled with SRM 1960 microspheres in water high **×** 45 mm wide **×** 20 mm thick. Input laser power to the sample was typically 60 to 80 mW at 620 nm and 90 to 120 mW at 570 nm with the Rhodamine 590 dye used. Wavelength scanning was accomplished with a piezoelectric inchworm micrometer which rotated a birefringent plate inside the dye-laser cavity, A complete spectrum was collected in about 20 min so that particle settling was not a problem.

The light scattered at 90° was detected with a silicon photodiode and a lock-in amplifier with output connected to a strip-chart recorder. Either the light intensity polarized parallel to the scattering plane (*I*_‖_) or that polarized perpendicular to the scattering plane (*I*_⊥_) could be detected by proper orientation of the collection arm and the polarizer (Fig. 12).

Since accurate alignment of the optics is critical to obtaining valid RLS spectra, a low-power He-Ne laser and a right-angle prism were used to carefully align the optical cell with both the incident dye laser beam and the collection arm. (Errors introduced by improper cell alignment are discussed in a later section.) Several additional considerations for obtaining valid RLS spectra from a microsphere suspension are discussed in Ref. [18].

### 4.2 Experimental Technique

Before loading the glass cell with particles, it was thoroughly washed using acetone and deionized water filtered through a 0.2 μm pore-size filter. The cell was then filled with filtered water, five drops of SRM 1960 were added, and the cell was ultrasonically vibrated to mix the particles and remove air bubbles.

To minimize multiple scattering, particle volume concentration was kept to about 15 ppm [19]. With a measured acceptance angle, *Γ*, of 0.8° the volume of particles sampled was about 27 mm^3^, so that with a 15 ppm concentration of particles, there were on average about 800 microspheres within the sampled volume. Although this is a relatively large number of sampled microspheres, a long (3 s) time constant was used on the lock-in amplifier to minimize the statistical fluctuation noise and to reduce Brownian motion noise. The Brownian motion of the particles was calculated to occur on a time scale of about 30 ms.

Five samples of SRM 1960, labeled R1, R2, R3-1, R3-2, and R3-3, were used in the RLS measurements. Samples R1 and R2 came from different vials of SRM 1960, while samples R3-1, R3-2, and R3-3 were all taken from a third vial. At least six RLS spectra, three *I*_‖_ and three *I*_⊥_, were taken for each of the five samples. After a spectrum was taken, peak wavelengths were measured from tic marks made at 10 nm intervals on the chart paper. Four peaks were measured in the *I*_‖_ spectra and three were measured in the *I*_⊥_ spectra.

### 4.3 Computer Analysis

**Fig. 13:**
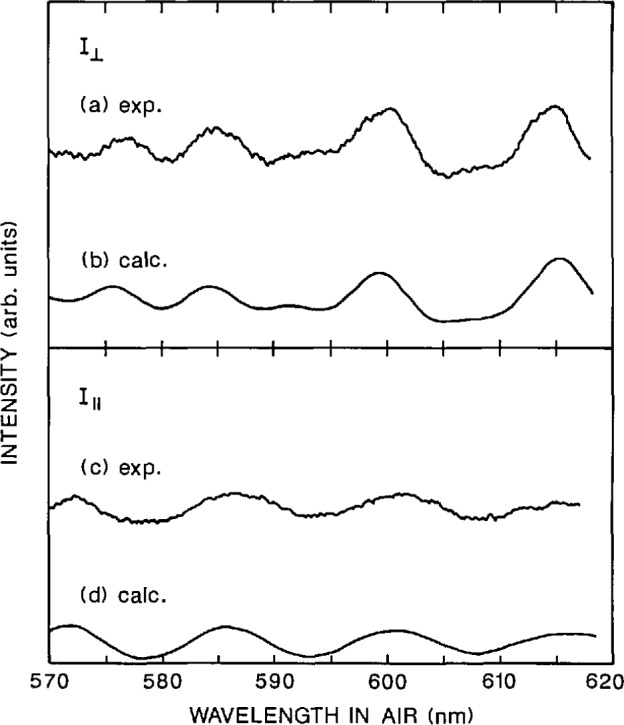
RLS spectra of SRM 1960 microspheres in liquid suspension (*I*_‖_ and *I*_⊥_.).

The calculated RLS spectra in Fig. 13 were generated on a CYBER 205 computer using a vectorized program based on Wiscombe’s Mie-scattering code [20]. The Mie intensities for a single dielectric sphere are [21]

$$\begin{array}{c} I_{⊥}(\theta ,x)=\frac{I_{0}}{k^{2}R^{2}}|\sum_{n=1}^{\infty }\frac{2n+1}{n(n+1)}[a_{n}\frac{P_{n}^{1}(\cos\theta )}{\sin\theta }\\ \;{+b_{n}\frac{dP_{n}^{1}(\cos\theta )}{d\theta }]|}^{2}, \end{array}$$ (6)

$$\begin{array}{c} I_{∥}(\theta ,x)=\frac{I_{0}}{k^{2}R^{2}}|\sum_{n=1}^{\infty }\frac{2n+1}{n(n+1)}[a_{n}\frac{dP_{n}^{1}(\cos\theta )}{d\theta }\\ \;{+b_{n}\frac{P_{n}^{1}(\cos\theta )}{\sin\theta }]|}^{2}, \end{array}$$ (7)

where *θ* is the scattering angle, *x = πd/λ* is the size parameter of the particle, *d* is the microsphere diameter, *λ* is the incident light wavelength in water, *I*_0_ is the intensity of the incoming beam, *R* is the distance from the particle to the detector. $P_{n}^{1}$ is an associated Legendre function, *k* = 2*π*/*λ* is the wavenumber, and *a_n_* and *b_n_* are the Mie scattering coefficients which are functions of *x* [21].

The wavelength dispersions of the refractive indices of polystyrene and water were taken into account using linear interpolation formulas from published data [22] over the wavelengths of interest (570 to 620 nm). (For the broad, collective resonance peaks of the present experiment, linear approximations were more than satisfactory, although they would not be for the sharp resonances of the individual microspheres.) If *λ* is in nanometers, then

$$n_{w}\approx 1.353-3.33\times 10^{-5}\lambda$$ (8)

and

$$n_{p}\approx 1.638-4.06\times 10^{-5}\lambda$$ (9)

are the refractive indices of water and polystyrene, respectively.

To account for the size distribution of the SRM 1960 microspheres, it was assumed that the diameters have a Gaussian distribution and that the suspended particles scatter independently. This allows integration over diameter and computation of an average light-scattering intensity:

$$\begin{array}{c} {\bar{I}}_{⊥,∥}(\lambda ,\theta )\approx \int_{d_{m}-\delta }^{d_{m}+\delta }d\xi \exp[-{\left(\xi -d_{m}\right)}^{2}/(2\sigma_{d}^{2})]\\ I_{⊥,∥}(\theta ,\pi \xi /\lambda ), \end{array}$$ (10)

where *d*_m_ is the mean diameter and on is the standard deviation of the size distribution. (Note that this expression ignores the small variation in scattered intensity with diameter; this will not affect the mean-diameter measurement.) The integration, which extends over 2*δ* = 6*σ*_d_, was carried out by computing the scattered intensity for an extended range of the size parameter (10,000 values) and then summing the appropriate values multiplied by the Gaussian factor. Typically, about 1/5 of the 10,000 values were included in each sum.

### 4.4 RLS Results

Representative RLS spectra for *I*_‖_ and *I*_⊥_ are shown in Fig. 13. Figure 13 shows a calculated spectrum for a collective sample of dielectric microspheres and an experimentally measured spectrum for a water suspension of SRM 1960 microspheres. From each of the experimental RLS spectra, the peak wavelengths were measured as noted earlier. Table 6 gives the measured wave-lengths for each of the five samples (Rl, R2, R3-1, R3-2, and R3-3); these values are the means from at least three RLS spectra.

A mean diameter for the SRM 1960 microspheres can be determined by RLS since the peak wavelengths in a collective spectrum vary almost linearly with particle diameter [18]. This is due to the fact that the frequency of a peak in a single-particle spectrum is a function of *d/λ* only, neglecting the (small) wavelength dispersion of the refractive indices. This near-linearity of *d* vs. *λ* for a collective spectrum permits an analytical best-fit diameter if the estimated diameter is close to the minimum of the square deviation, *Q* (*d*):

$$Q(d)=\sum_{i=1}^{8}{\left[\lambda_{i}^{m}-\lambda_{i}^{\mathrm{th}}(d)\right]}^{2},$$ (11)

where $\lambda_{i}^{m}$ represent the seven measured peak wavelengths and $\lambda_{i}^{\mathrm{th}}$ represent the corresponding calculated peak wavelengths (four *I*_‖_ peaks and three I_⊥_. peaks). Using the calculated peak wave-lengths $\left(\lambda_{i}^{0}\right)$ for a diameter (*d*_mo_) near a minimum of Eq. (11) and invoking the (near) proportionality between *λ* and *d*, the diameter *d*_min_ which corresponds to the minimum *Q* can be computed by taking the derivative of Eq. (11) and setting it equal to zero. The resulting expression for the best-fit diameter is:

$$d_{\min}=\frac{\sum \lambda_{i}^{m}\lambda_{i}^{0}}{\sum {\left(\lambda_{i}^{0}\right)}^{2}}d_{\mathrm{mo}}.$$ (12)

It should be noted that the *Q* vs. *d* curve has a series of near-periodic local minima [18], so that the above procedure yields, in general, a series of “best-fit” diameters, one at each local minimum. This would make it difficult to match the peaks in the RLS spectra of single microspheres, in which there are many peaks of different polarization and order [16]. However, peak assignment is much simpler for the collective RLS spectra of the present experiment since there are far fewer, and much broader, peaks. Thus, a unique best-fit diameter can be easily obtained. Starting from the CDF result of 9.89 μm as the value for *d*_mo_, a least-square diameter was determined for each of the five samples of SRM 1960; the results are summarized in Table 6. The within-vial agreement for samples C1, C2, and C3 was better than 0.001 μm, or 0.01% of the mean diameter. The other results, for samples A and B, differed from this value by a detectable amount. This may be evidence for a small amount of vial-to-vial variability in mean diameter of SRM 1960, although this possibility was not pursued.

The RLS-determined diameter of the SRM 1960 microspheres is taken to be the mean of the five values or 9.898 μm.

### 4.5 RLS Error Analysis

There are several sources of random and systematic error in the RLS technique. The most significant random errors are in the measurements of peak wavelength and scattering angle. The biggest contributions to the systematic uncertainties are in refractive index, peak wavelength, scattering angle, acceptance angle, and intensity variation of the laser beam. Possible error sources that were assumed to be small and were therefore neglected are: multiple scattering, sampling, polarization misalignment, backscattering, agglomeration, particle inhomogeneities, particle asphericity, and temperature effects.

#### 4.5.1 Random RLS Errors

The 3*σ* random error, *R*, determined from the five diameter measurements in Table 6 is ± 0.0076 μm, and this value is used in Eq. (13) to calculate the total uncertainty of the RLS measurements. Various potential sources of this random error are discussed below.

##### Wavelength

Random errors in the measurement of the peak wavelengths are the irreproducible variations in locating the peak of a broad resonance. This, in turn, is due to the width of the resonance, to noise on the experimental RLS spectra, and to random nonlinearities in the wavelength scanning. The specified accuracy of the wavelength meter is better than 1 part in 10^5^, so that random and systematic errors in the wavelength meter can be safely ignored.

The random wavelength error was determined by calculating the standard deviation of the peak wavelengths from the three repeat RLS spectra taken on each sample. This was done for all peaks in all the spectra, and a mean taken of these numbers; the result was 3*σ_λ_* =0.45 nm. Since the measured wavelengths are all approximately 600 nm, this gives a random diameter uncertainty of ±0.008 μm per measurement, or ±0.004 μm for all five RLS measurements.

##### Scattering Angle

The random component of the scattering angle error is due to slight, irreproducible misalignments of the optical cell when it is repositioned between spectra. To determine the effect on diameter measurement due to this error, RLS spectra were calculated for *θ* = 89° to 91° in 0.1° intervals, and a best-fit diameter was determined at each of these angles. The variation in diameter was only about 0.01 μm for the 1° change in scattering angle. Thus, using an estimated maximum random angle error of ± 1°, the error due to random misalignment of the optical cell is calculated to be about ±0.01 μm per measurement, or ±0.004 μm for all five measurements.

##### Total Random Error

Summing the above two contributions in quadrature gives a total random uncertainty of ± 0.0064 μm, in good agreement with the 3*σ* random uncertainty (±0.0076 μm) calculated from the five RLS measurements.

#### 4.5.2 Systematic RLS Errors

##### Refractive Index

Uncertainty in the refractive index of polystyrene is the largest source of error in the RLS measurements. As discussed in Refs. [23] and [24], the values for *n*_p_ for individual 1 μm diameter polystyrene spheres ranged from 1.577 to 1.595 (at *λ* =632.8 nm) when measured by different researchers. Several possible explanations have been proposed for this spread in values including: experimental uncertainties when measuring single particles; surface coatings on dried particles; and real differences in optical properties from particle to particle. In the present study, the bulk value for *n*_p_ was used because the RLS spectra average over many particles, eliminating possible differences between particles, and because the SRM 1960 microspheres are much larger than the 1 μm spheres mentioned above, minimizing possible size effects. The bulk index was taken to be 1.588 (at *λ* =632.8 nm), with an uncertainty of ± 0.001 and a V-number dispersion of 30.8, as measured by Matheson and Saunderson [25].

In addition to *n*_p_ error, there are also small errors resulting from the use of linear dispersion relations for *n*_p_ and *n*_w_ and from uncertainties in the bulk value of *n*_w_. These are estimated to give, at most, another 0.0005 systematic error in the refractive index. Thus, the total refractive index error is 0.0015, which results in a systematic diameter uncertainty of ±0.01 μm.

##### Wavelength

As noted earlier, the systematic error in the wavelength meter is negligible. However, this assumes static conditions, i.e., that the laser wave-length is not changing. Because the wavelength is, in fact, continuously changing during an RLS spectrum measurement, a systematic offset results from the 2 s time constant of the wavelength meter and the 3 s time constant of the lock-in amplifier. Since the wavelength scan rate in the RLS measurements was 0.05 nm/s, the combined time constant of 5 s results in a maximum systematic offset error of 0.± 25 nm in wavelength or ± 0.004 μm in diameter.

##### Scattering Angle

Since the scattering angle, *θ*, was experimentally set by autocollimating the incident laser beam from two (nominally) orthogonal sides of the optical cell, systematic scattering-angle errors may arise from two different sources: (i) inaccuracies in the optical cell angles and (ii) systematic misalignment of the optical cell.

**Fig. 14:**
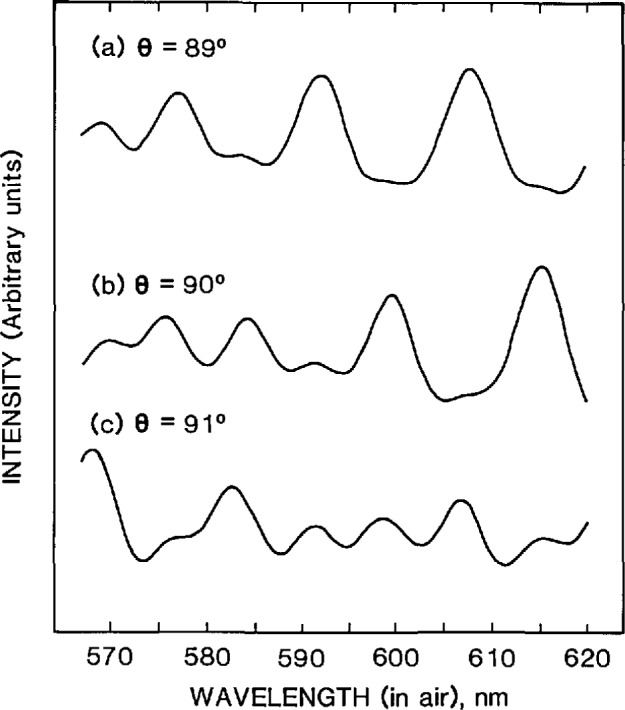
Calculated RLS spectra for 89°, 90°, and 91°.

To measure this component of error, the alignment helium-neon laser beam was autocollimated off all four sides of the cell and the maximum beam offset determined. The angle error measured in this manner was about ± 0.5°. There are other indications that the systematic angle errors were less than 1°; these come from inspection of Fig. 13, viz., the good agreement between the experimental RLS spectra and the spectra calculated for exactly 90°. Note that although the peak wavelengths do not change much with angle, the peak amplitudes do change dramatically as a function of angle (Fig. 14). The excellent match in peak amplitudes between calculation and experiment is a good indication that *θ* was very close to 90°. In addition, for *θ*’*s* other than 90°, the peaks that were suppressed at 90° make significant contributions to the spectra (Fig. 14). No such extraneous peaks were seen, further evidence for a scattering angle very close to 90°.

Taking the systematic angle error to be at most ±0.5° results in an upper limit of ±0.005 μm on the diameter uncertainty.

##### Acceptance Angle

In addition to the scattering-angle uncertainty, there is an error component due to the finite acceptance angle, *Γ*, of the collection optics [18]. This angle was measured to be ±0.8° at the half-transmission points. Using this value as input, calculations were performed in which 1/2 of the intensities at 90.4° and 89.6° were added to the intensity at 90°. The resultant shift in the peak wavelengths was less than ± 0.06 nm, giving a systematic diameter uncertainty of ± 0.001 μm.

##### Laser Intensity Variation

Because of the wave-length-dependent properties of the laser intensity stabilizer, the intensity that was incident upon the microsphere suspension was not constant, but instead increased by about 50% as the dye laser scanned from 620 to 570 nm. Typical laser powers were 60 to 90 mW at 620 nm, and 90 to 120 mW at 570 nm. The effect of this input power rise was simulated in computer calculations by including a linear intensity factor in Eq. (10). The resulting shift in peak wavelengths was less than ±0.1 nm, for a maximum diameter uncertainty of ±0.002 μm.

#### 4.5.3 Total Diameter Uncertainty from RLS

The above errors in the RLS measurement are summarized in Table 7. They can be combined to get the total error by using the expression

$$\begin{array}{l} U_{T}=R+U_{S}\\ \;=R+|\delta_{n}|+|\delta_{\lambda }|+|\delta_{\theta }|+|\delta_{\Gamma }|+|\delta_{I}|, \end{array}$$ (13)

in which *R* and *U*_S_ are the total random and total systematic components, respectively, of the measurement error. The systematic errors in diameter measurement are: *δ_n_*, refractive index uncertainty; *δ_λ_*, peak wavelength uncertainty; *δ_θ_*, scattering-angle uncertainty; *δ_Γ_*, finite-acceptance-angle error; and *δ_I_*, an error due to a linear rise in the laser intensity as the wavelength was varied.

Substituting the various error values into Eq. (13) gives a total uncertainty of ±0.03 μm at the 99% confidence level (3*σ*), for the RLS-determined mean diameter of SRM 1960.

### 4.6 Final Results of the RLS Measurements

The mean diameter of SRM 1960 determined from resonance light scattering is 9.90 ± 0.03 μm.

## 5. Summary and Conclusions

The results for the mean diameter of the SRM 1960 microspheres, as determined by all three micrometrology techniques, are summarized in Table 8. The agreement between the measurements is excellent, well within the stated uncertainties of the techniques: this study is probably the most accurate intercomparison of its type ever made in the dimensional metrology of microspheres.

It is significant that all of the measurements of SRM 1960 were made on the particles in different physical environments, under different measurement conditions, and using different physical principles to determine the mean particle size. With center distance finding, the microspheres were measured dry, in air. By comparison, with metrology electron microscopy they were measured dry and in an ultrahigh vacuum and, moreover, were irradiated with relatively high-energy electrons (30 keV). Finally, with resonance light scattering, the SRM 1960 microspheres were suspended in water at room temperature and pressure. Despite these environmental differences, the excellent agreement between all three measurements is evidence that the physical principles of the measurements are understood and that major systematic errors were accounted for. The mutual agreement is also an indication that the polystyrene microspheres of SRM 1960 are not significantly affected by their environment, at least for those used here. In particular, there was no evidence for particle outgassing in an ultra-high vacuum.

In summary, SRM 1960 is an accurately calibrated standard artifact for micrometrology applications that is highly uniform in size and shape and is dimensionally stable under reasonable changes in environment. It should find many uses in industry, technology, and basic research.

## Figures and Tables

**Table 1:** Results from center distance finding

Vial No.	Sample No.	Photos taken	Spheres measured	Outliers		Diameter (μm)	
				Over	Under	Mean	Median
1	C1	20	1074	11	0	9.891	9.892
1	C2	2	132	1	0	9.912^a^	9.905^a^
1	C3	2	107	2	0	9.890	9.888
2	C4	4	265	0	0	9.889	9.888
3	C5	6	239	3	0	9.892	9.878
4	C6	4	224	2	0	9.893	9.884

Combined		38	2041	19	0	9.891^b^	9.886^b^
						3*σ_n_*_−1_= 0.0047^b^	0.016^b^

a Statistical outliers.

b These values do not include the statistical outliers.

**Table 2:** CDF error budget^a^

Measurement	Error source	Random error (μm)	Systematic error (μm)
CD measurement	Film stability and readout	0.08	
	Magnification scatter	0.02	
	Sphere flattening at contact		0.002
Sampling (*n* ≈ 2000)		0.27	
Off-Axis magnification	Measuring off-axis magnification (make overlay) (use overlay)	0.02	0.01
On-Axis magnification	Stage micrometer (SM) calibration		0.003
	SM image-length readout		0.006
	SM image-length correction		0.006
	Magnification scatter		0.009

Total error per measurement		0.28	0.036
Total error on *d*_m_		0.006	0.036

a The errors are for a single center-distance measurement.

**Table 3:** Repeat MEM measurements from five microspheres in sample M1^a^,^b^

Particle #	*d*_1_	*d*_2_	*d*_3_	*d*_1_*−d*_3_	*σ_n_−*1
1	9.890	9.875	9.859	0.031	0.016
2	9.859	9.811	9.796	0.063	0.033
3	9.875	9.827	9.827	0.048	0.028
4	9.875	9.859	9.811	0.064	0.033
5	9.811	9.764	9.764	0.047	0.027

Mean	9.862	9.827	9.811	0.051	

a All measurements are in μm.

b The *σ_n_−*1 are the standard deviations of the three measurements.

**Table 4:** Results from metrology electron microscopy

Sample	*N*	*d* (μm)	*s*_m_^a^ (μm)
M1	28	9.884	0.013
M2	28	9.881	0.014
M3	25	9.894	0.020

Combined	81	9.886	0.016
		3*σ_n_*_−1_ = 0.018 μm	

a *s_m_* is the standard error on the mean of the *N* measurements in each sample.

**Table 5:** MEM error budget^a^

	Error source	Random (μm)	Syst. (μm)
Random	Sampling	0.03	
	Spatial resolution	0.003	
	Cosine error	0.003	
Systematic	Interferometer least count		0.016
	Stage travel digitization		0.02
	E-Beam erosion		0.01

Total error		0.03	0.046

a The errors are for all 81 MEM measurements.

**Table 6:** Peak wavelengths and diameters from RLS^a^

Sample						
	R1	R2	R3-1	R3-2	R3-3	
*I*_‖_	576.8 nm	575.7 nm	576.2 nm	576.5 nm	576.6 nm	
	584.1	583.3	584.2	584.3	584.6	
	599.3	599.4	599.9	599.6	599.5	
	615.2	614.7	614.2	614.3	614.0	
*I*_⊥_	572.0	571.9	572.4	572.1	572.1	
	586.0	586.1	586.1	586.3	586.4	
	601.5	601.2	601.1	600.7	600.9	

*d*_min_, μm	9.901	9.894	9.899	9.898	9.898	3*σ_n_*_−1_ = 0.0076 μm
*Q*, nm^2^	3.8	8.0	5.8	5.8	29.9	

a All wavelengths are the means of data from at least three RLS spectra.

**Table 7:** RLS error budget^a^

	Error source	Random (μm)	Syst. (μm)
Random	Wavelength	0.004	
	Scattering angle	0.005	
Systematic	Refractive index		0.01
	Wavelength		0.004
	Scattering angle		0.005
	Acceptance angle		0.001
	Intensity		0.002

Total error		0.006	0.022

a The errors are for all five RLS measurements.

**Table 8:** Summary of results for mean diameter of SRM 1960

Technique	*d*_m_, μm	Unc., μm
CDF	9.89	±0.04
MEM	9.89	±0.06
RLS	9.90	±0.03

## Footnotes

2 Certain commercial equipment, instruments, or materials are identified in this paper to specify adequately the experimental procedure. Such identification does not imply recommendation or endorsement by the National Institute of Standards and Technology, nor does it imply that the materials or equipment identified are necessarily the best available for the purpose.

## 6. Appendix A. Microscope Calibration for Image Magnification and Distortion

In this Appendix, a description is given of the microscope calibration process. The commercial microscope calibrated in this manner had a 20 ×, 0.50 N.A. objective, a 2.0 × relay lens, and a 12.5 × photo eyepiece.

First, one row of a hexagonal array of 10 μm SRM 1960 microspheres is positioned so as to cross the center of the microscope field of view (FOV), and its focal spots are photographed. Next, the microscope slide is shifted in-line by three sphere diameters and photographed again. The distances between adjacent sphere centers are measured in both photographs, and changes in them are plotted (Fig. 6a). If image distortion is present, these systematic length changes, which are proportional to the object shift, will also depend on the initial position of each sphere pair.

The data points in Fig. 6a are then used to find the accumulated length changes when a line segment of length *d* (= one sphere center distance) is shifted in-line from one side of the FOV to the other, moving in steps 3*d* long and starting at the far left position (indicated by circles). The process is repeated starting at the second left position, then again at the third. The result is three groups of data points which are shifted vertically as a group relative to each other until they fall into a best fit on a common curve (Fig. 6b). This curve represents, in relative terms, the change in film scale when moving along a FOV diameter. In other words, the curve represents the image magnification in terms of its on-axis value (the scale distortion). A graphic integration of Fig. 6b shows how much a point image will be displaced from its nominal position due to the variation of magnification across the FOV (this is the image distortion).

Next, a section of a calibrated stage micrometer is positioned so as to almost fill the FOV (to maximize data resolution), and then it is photographed. The image length is measured and corrected for image distortion using Fig. 6c. The corrected length now yields the on-axis value of the image magnification. Combined with the data in Fig. 6b, one now has the complete magnification curve for the combination of microscope objective, relay lens (if any), and projection eyepiece that was used.

The justification for the above calibration procedure can be found by considering that the image distortion of well centered, high quality microscope optics is not a function of orientation in the FOV, but only of the off-axis distance. Its derivative with respect to radius represents the scale distortion (Fig. 6b), and its second derivative shows by how much a line segment will change if it is radially shifted from a selected off-axis point (Fig. 6a). Adopting a 3*d* shift rather than a 1*d* shift results in better data resolution in Fig. 6b. However, this did not result in an averaging over three center distances, only over one.
